# S56. PHOENIX GROUP, A PROJECT TO PREVENT RELAPSES IN SCHIZOPHRENIA

**DOI:** 10.1093/schbul/sby018.843

**Published:** 2018-04-01

**Authors:** Marcelo Chiramberro, Tuula Kieseppä, Asko Wegelius, Tuomo Töhönen, Jenna Ilvonen, Heli Väisänen, Katja Sarkkinen, Jenni Tikkala, Marita Leiponen, Markku Ruppa, Matias Hyytiäinen, Ira Hynninen-Sundelin, Esa Anttonen

**Affiliations:** 1 Peijas Hospital, University of Helsinki; 2 Helsinki University Hospital

## Abstract

**Background:**

Schizophrenia is a syndrome of variable and highly disruptive psychopathology that infers emotion, perception, and several aspects of behavior. Relapses caused by noncompliance are common and may lead to hospitalizations. In many ways they increase social disability and health care costs.

The project’s philosophy was based on the motivation of the patients to continue their treatment, improve their quality of life, and reduce disease relapses, which in turn reduces public health care costs.

**Methods:**

We enrolled 10 schizophrenia and schizoaffective patients with a poor treatment adhesion in a pilot group. All the patients were at the acute psychosis ward. The patient’s average hospitalization days were 180 days/year, and they were highly noncompliant with the open ward treatment accompanied with assertive community treatment model. Seven patients had depot injections and three patients had oral antipsychotic medication.

During the hospital treatment the patients started in a group, which continued after the discharge. Weekly group meetings with different activities were organized at the hospital by the ward personnel. The doctor, nurses, a psychologist and an occupational therapist participated the group with different combinations and in a nonhierarchical manner. Psychoeducation was used to increase the knowledge and coping with the disorder. Functional group activities like cooking, arts and visits to different places were organized.

The clinical parameters including the work status, relapse rates and hospitalization days were evaluated at every 6-months during 18 months. For the clinical measurements we used the Brief Psychiatric Rating Scale (BPRS) and 15D Health Related Quality of Life instrument.

**Results:**

At 18 months follow-up, eight of 10 group members had not needed hospitalization at all, one needed hospitalization of 15 days and another 20 days. Both of them were in voluntary treatment. During the pilot stage, two patients got jobs.

At five year follow-up, five of 10 initial patients were full-time employed persons. None have needed hospitalization after 18 months.

**Discussion:**

The intensive group focused on noncompliant patients, organized by the hospital ward, where these patients had been recurrently treated, improved substantially the patients’ commitment to the treatment and decreased rehospitalizations. The non-hierarchical group operating in the interface of the hospital and open ward was able to cause a significant reduction of general health costs and an improvement in the quality of life of these patients. They were reintegrated into society, and the stigma and marginalization associated with psychoses decreased while the self-esteem improved. The patients were able to create friendships with others in the same situations. They helped one another, and thus also improved their own self-help capacity. These elements prevent social isolation, treatment nonadherence and functional deterioration, which also would be a risk for increased violence and suicide.

The group meetings began during the hospital treatment, and the patients intensively continued in the familial group after the discharge. However, the goal for these patients is gradually to leave the group and attend other open-ward, occupational and social activities.

**Conclusions:**

Through psycho-educational interventions combined with pharmacological and psychological treatments, Phoenix group, a patient orientated, peer supporting and hospital wall-breaking method obtains results clearly observable that we can warmly recommend.

